# Human Plasma Causal Immunome-Phenome Mapping Reveals Cardiovascular Enrichment and Therapeutic Target Opportunities

**DOI:** 10.21203/rs.3.rs-11108738/v1

**Published:** 2026-10-21

**Authors:** Shila Taslima, John Zhang, Huilan Tan, Yanwen Liu, Dhrubo Ahmad, Martin Young, Suresh Verma, Min Xie, Jianli Zhao, Yajing Wang

## Abstract

Background Circulating immune proteins represent an important class of therapeutic targets, but existing proteome-wide genetic studies have not systematically resolved immune-related target–disease relationships across a broad disease phenome within a unified framework that integrates causal inference, locus colocalization, orthogonal protein-level support, independent replication, and standardized evidence grading. Methods We developed the Human Plasma Immune Atlas (HPIA), a tiered proteogenomic framework built from public datasets. We curated 1,007 plasma immune proteins from 2,923 Olink Explore analytes, instrumented each encoding gene with its strongest blood cis-expression quantitative trait locus (eQTLGen), and tested effects on 2,469 FinnGen R12 disease endpoints by Mendelian randomization (MR), followed by colocalization, protein-level validation with INTERVAL cis-protein quantitative trait loci, 2-population replication in UK Biobank, and prespecified 5-tier evidence grading. A companion Plasma Immune Risk Score (PIRS; elastic-net Cox) is released for predicting targets. Results Across 1,656,872 evaluable gene–disease tests, 1,016 associations involving 220 immune genes and 381 diseases met FDR < 0.05, of which 417 showed strong evidence of colocalization (PP.H4 ≥ 0.80). Positive-control analyses recapitulated five established immune drug-target relationships and the ACE–hypertension relationship with directionally concordant genetic effects. Among 189 discovery associations evaluable in UK Biobank, 162 (86%) showed concordant effect directions and 89 met the prespecified replication criterion. Cardiovascular phenotypes contained the largest number of high-confidence associations, accounting for 67 of 176 core target–disease pairs, while hypertension was associated with 28 immune proteins. Integration of MR, colocalization, protein-level evidence, and replication promoted five target–disease pairs to the highest evidence tier, including ANXA2-hypertension and PPP3R1-venous/lymphatic disease. Systematic prioritization further identified 45 colocalized candidate therapeutic target–indication pairs with genetically inferred directions of intervention. In validation evaluation, the PIRS achieved a cross-validated concordance index of 0.777. Conclusions HPIA provides an open, genetics-anchored map connecting the circulating immune system to the human disease phenome and distinguishes initial genetic associations from target–disease relationships supported by orthogonal evidence. The framework identifies cardiovascular phenotypes as a major concentration of genetically supported immune associations and provides experimentally testable hypotheses for therapeutic target discovery, drug repurposing, and immune-informed disease stratification.

